# Biomimetic recombinant of red blood cell membranes for improved photothermal therapy

**DOI:** 10.1186/s12951-021-00949-7

**Published:** 2021-07-18

**Authors:** Pengkai Wu, Xing Jiang, Shuai Yin, Ying Yang, Tianqing Liu, Kaikai Wang

**Affiliations:** 1 School of Pharmacy, Nantong University, 226001 Nantong, China; 2 Department of Hepatobiliary Surgery, the Affiliated Drum Tower Hospital of Nanjing University Medical School, 210093 Nanjing, China; 3 College of Nursing, Nanjing University of Chinese Medicine, 210029 Nanjing, China; 4 NICM Health Research Institute, Western Sydney University, 2145 Westmead, Australia; 5 Nantong Municipal Hospital of Traditional Chinese Medicine, 226001 Nantong, China

**Keywords:** Drug Delivery, Red blood cell membrane, Disassembly-Reassembly, IR780, Photothermal Therapy

## Abstract

**Background:**

RBC membrane derived nanoparticles (NPs) represent an emerging platform with prolonged circulation capacity for the delivery of active substances. For functionalize derived RBCs NPs, various strategies, such as biomimetic rebuilding of RBCs, chemical modification or inserting ligands, have been carried out to improve their performance. However, one potential adverse effect for these methods is the structural failure of membrane proteins, consequently affecting its original immune escape function.

**Results:**

In this study, we reported a green technology of “disassembly-reassembly” to prepare biomimetic reconstituted RBCs membrane (rRBCs) by separating the endogenous proteins and lipids from nature RBC membrane. IR780 iodide was used as a pattern drug to verify the property and feasibility of rRBCs by constructing IR780@rRBC NPs with IR780@RBC NPs and free IR780 as controls. The results demonstrated the superiority of IR780@rRBC NPs in toxicity, stability, pharmacokinetics and pharmacodynamics compared with IR780@rRBC and free IR780.

**Conclusions:**

The reported “disassembly-reassembly” strategy shows great potential to produce controllable and versatile rRBC membrane-inspired delivery platform, which may be used to overcome the deficiency of functionalization in cell membrane coated nanoparticles .

**Graphic abstract:**

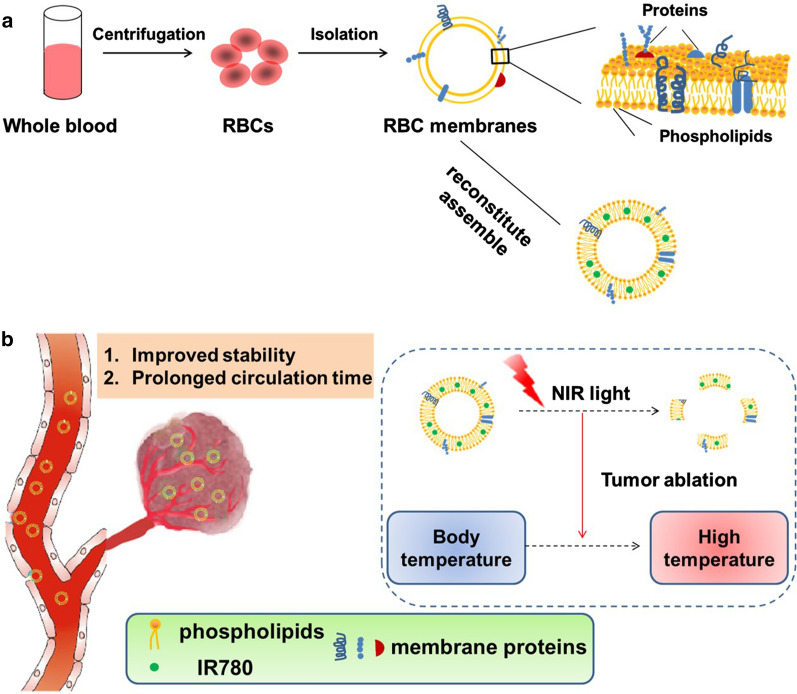

**Supplementary Information:**

The online version contains supplementary material available at 10.1186/s12951-021-00949-7.

## Background

Over the past few decades, red blood cells (RBCs or erythrocytes) based drug delivery systems have attracted considerable attention to improve the pharmacokinetics, biodistribution and pharmacodynamics of therapeutics or diagnostics [1, 2]. Commonly, administration of most therapeutic agents by conventional formulations and dosage may lead to the limited clinical applications and therapeutic index, which are due to their rapid clearance and potential immunogenicity in vivo [3, 4]. In contrast, inherent biochemical and biophysical properties of erythrocytes make them a significant advantage over alternative technologies in terms of half-life, stability, versatility, safety and ease of manufacture as an ideal drug delivery platform [5–7]. Therefore, a variety of substances (e.g. enzymes, glucocorticoid analogs, immunosuppressant drugs, etc.) have been tried to be loaded in the erythrocytes for modulating their pharmacokinetics, and the encapsulated formulations also proved to be safe and to deliver the promise of a new technology capable of convenient solutions for old dilemmas.

Notably, numerous attempts have been tried to develop RBC membranes derived nanoparticles by utilizing its functional features. For example, various nanoparticles, including polymeric nanoparticles [8], Fe_3_O_4_ nanoparticles [9], mesoporous nanoparticles [10], upconversion nanoparticles and gold nanocage and etc. [11, 12], have been coated with RBC membranes and then inherit its immune system evade ability to achieve long term circulation in blood. Further, RBC membranes functionalization have been carried out to improve the properties of native RBC membranes derived nanoparticles, such as deformability, stability, targeting and circulation abilities. The strategies mainly include biomimetic rebuilding of RBCs, chemical modification on the membranes surface or inserting targeted ligand-linker-lipid conjugates into the membranes [13, 14]. One potential adverse effect for these methods is the structural failure of membrane proteins, such as the self-recognition marker CD47 of preventing phagocytosis by macrophages, consequently affecting its original immune escape function [15, 16].

A key concept for preserving structural integrity is green technologies, which are defined as the elimination of hazardous substances and prevention of health impacts in the design process [17–19]. With utilizing this technology, the fabricated biomaterial system was expected to restore the natural structure to avoid membrane proteins damage. For example, Wang and co-workers reported a reconstituted lipoprotein nanoparticle (RLNs) using whole-components of endogenous lipids and native apolipoproteins to replace purified apolipoprotein and commercial lipids [20]. Inspired by the above pioneering studies wherein reconstituted structure was created via a green strategy of “disassembly-reassembly”, we endeavored to construct a reconstituted RBCs (rRBCs) mimic that composed of isolated lipids and original proportions of endogenous proteins for reassembly, and finally possessed the complete features of native RBCs.

**Fig. 1:**
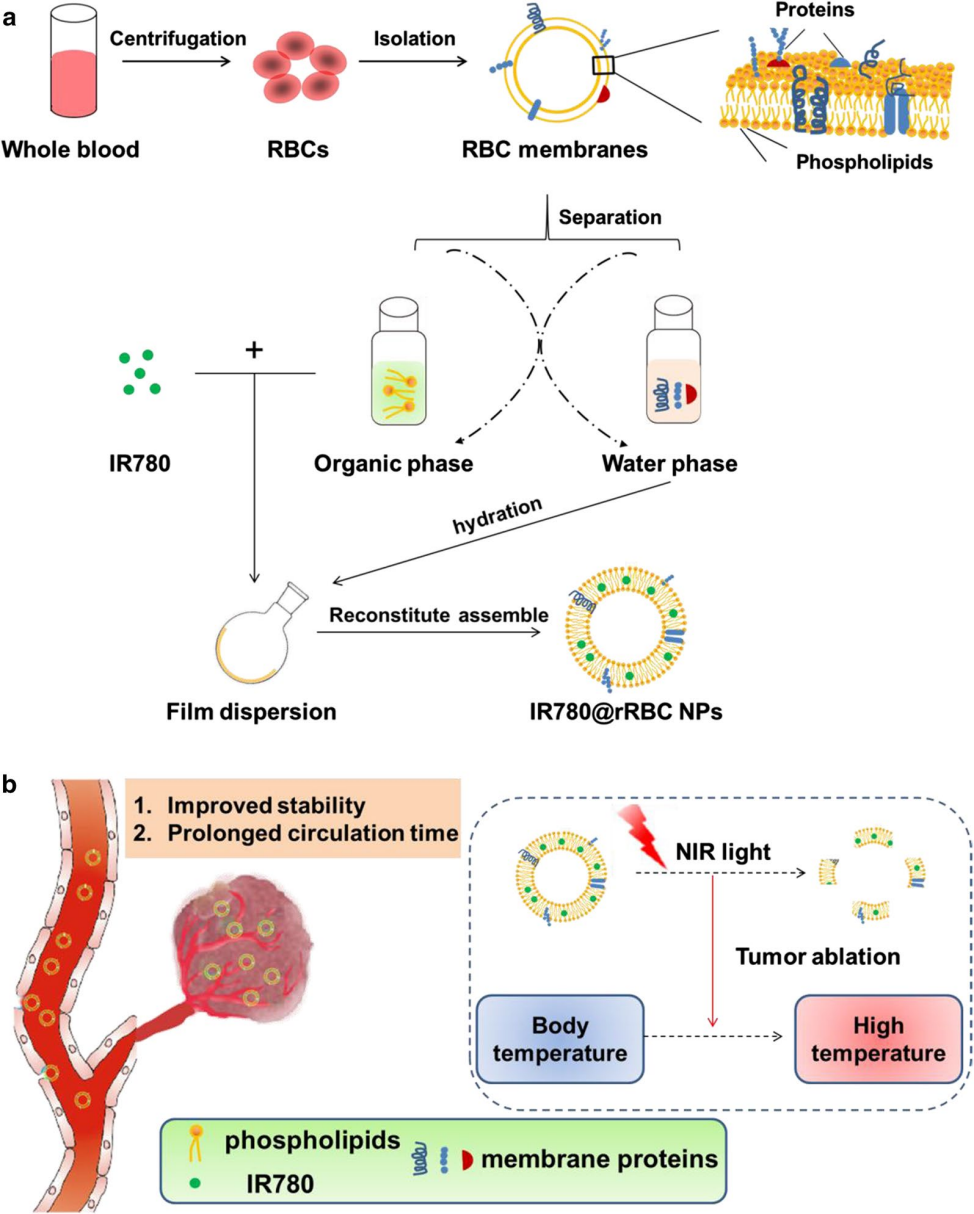
The scheme and procedure for the preparation of IR780 loaded reconstitute RBC membrane nanoparticles (IR780@rRBC NPs). **A** RBC membrane was prepared by a hypotonic method. Then RBC membranes were treated by mixed organic solvent, to separate the lipids and proteins. Lipids part was used to load IR780 by film dispersion method. At last, IR780@rRBC NPs were form by adding proteins with the film, following further extrusion. **B** IR780@rRBC NPs increased stability in vitro, and prolonged circulation capacity and enhanced PTT therapeutic efficiency in vivo

A pattern drug for demonstrating the above strategy in our biomimetic rRBCs is near-infrared (NIR) dye of IR780, which is a small organic molecules that absorb radiation in the wavelength range 700–1000 nm [21]. In the treatment of tumor, IR780 has been reported that it is a good candidate for the photodynamic therapy, photothermal therapy and NIR imaging due to its capability of generating a singlet oxygen, high photothermal conversion efficiency and preferential accumulation in multiple tumor cells [22]. However, strong hydrophobicity and high toxicity of IR780 limit its further clinical applications [23]. Here, exploiting feasibility of this novel biomimetic green strategy of “disassembly-reassembly”, IR780 loaded rRBCs nanoparticles (IR780@rRBC) was fabricated with IR780@RBC and free IR780 as controls. The construction process of IR780@rRBC nanoparticles was as shown in Fig. 1: ①RBCs membrane was first isolated and purified from nature RBCs; ②endogenous lipids and proteins were separated from the obtained RBCs membrane; ③the separated lipids were used to encapsulate IR780 by film dispersion method, ④and the original proteins of RBCs membrane were added to the film by hydration method for reassembly of IR780 loaded rRBCs nanoparticles (IR780@rRBC).

In this study, we report and compare the solubility, toxicity, pharmacokinetics and pharmacodynamics of free IR780, IR780@RBC and IR780@rRBC. We expect the IR780@rRBC NPs formed by the above controllable and green assembly strategy to exhibit the superior performance in pharmacodynamics over IR780@RBC formed by a simple ultrasound method. And we secondly hypothesis that IR780@rRBC can improve the stability of IR780@RBC, and further contributing to the enhanced pharmacokinetics in vivo due to the prolonged circulation time. Based on these results, this controllable and green assembly technology will have variable applications in the functionalization of rRBCs derived nanoparticles because functional lipids can mix with isolated lipids in the reassembly process. Overall, rRBCs biomimetic show great potential to produce controllable and versatile RBC-inspired delivery platform for functionalize derived RBCs NPs in future study.

## Materials and methods

### Materials

IR780 was purchased from Sigma-Aldrich (St Louis, MO, USA). The mouse colon cancer cell line (CT-26) was purchased from Shanghai Institute of Cell Biology (Shanghai, China). DAPI was purchased from Beyotime Institute of Biotechnology (Shanghai, China). The rabbit CD47 antibody were obtained from Cell Signaling Technology. All other reagents were from Nanjing Wanqing Chemical Glassware Instrument unless otherwise stated.

### RBC membrane derivation

RBC membranes were obtained from nature RBCs following previously published protocols with modifications [24]. Briefly, whole blood was withdrawn from Balb/c mice and was centrifuged at 2000 rpm for 10 min at 4 °C to collect the erythrocytes, which were continually washed with ice cold 1×PBS for three times. Then, the washed RBCs subjected into a hypotonic treatment with 0.25×PBS in ice bath for 20 min, and subsequently centrifuged at 8000 rpm for 15 min to remove the released hemoglobin. Finally, the obtained RBC membranes were washed with 1×PBS twice and were centrifuged at 12,000 rpm for 10 min to get the RBC membrane.

### Preparation of IR780@RBC and IR780@rRBC

IR780@RBC was prepared by an ultrasonic method. Briefly, the obtained RBC ghosts (1 mL whole blood) were sonicated for 10 min in a bath sonicator (50 kHz, 100 W). The resulting vesicles were subsequently extruded serially through 400-nm and then 220-nm polycarbonate porous membranes. Then the resulted RBC-Membrane-Derived Vesicles were mixed with IR780 ethanol solution (1 mg/mL) and sonicated again for 5 min. Then the final product IR780@RBC was obtained by extruding through 220 nm polycarbonate porous membranes to remove free dye and dialyzing against water to remove ethanol.

IR780@rRBC was prepared by a film dispersion method [25]. In brief, the dehydrated lipids (1 mL whole blood) and 1 mg IR780 were dissolved in 5 mL of ethanol and the organic solvent was evaporated under vacuum at 37 ℃. The dried lipid film was then hydrated with 2 mL of endogenous proteins solution (1 mL whole blood) at 37 ℃ for 30 min and sonicated at 100 W for 5 min. Then the final product IR780@rRBC was obtained by extruding through 220 nm polycarbonate porous membranes to remove free dye. Endogenous proteins and dehydrated lipids were isolated and purified from RBC ghosts using an organic solvent method similarly with the purification method from human plasma sample [26, 27]. Briefly, the obtained RBC ghosts were dissolved in PBS buffer (pH 5 ~ 6) and delipidated by adding equal volume of ethanol. After centrifugation, the supernatant was mixed with equal volume of ethanol and centrifuged again. The final supernatant, which contained endogenous proteins and dehydrated lipids, was concentrated by polyethylene glycol (MW: 20,000) and dialyzed against PBS buffer, followed by precipitating dehydrated lipids with cold ethanol.

The preparation process of IR780@Liposome was similar to that of IR780@rRBC by using a film dispersion method. Briefly, 20 mg of Egg PC and 1 mg of IR780 was dispersed in 5 mL ethanol, followed by evaporation for 30 min. After that, the dried lipid film was hydrated (2 mL distilled water), sonicated and extruded using the same condition as above to obtain IR780@Liposome system.

SDS-PAGE was used to confirm the protein integrity of RBC membranes in IR780@RBC and IR780@rRBC nanoparticles. Western blotting was conducted to assess the presence of CD47 protein. The amount of the IR780 in IR780@RBC and IR780@rRBC was determined by UV-VIS-NIR absorption spectra (UV1800, MAPADA) according to standard curve. IR780 encapsulation efficiency was calculated as follows:

$${\text{Encapsulation efficiency}}({\%}) =\,{{\text{weight of IR780 in NPs}}} / {{\text{weight of total added IR780}}} \times 100{{\% }}$$

### Characterization and stability of IR780@rRBC

Structure was examined using a transmission electron microscope (TEM, H-600, Hitachi, Japan). Particle size was determined by dynamic light scattering (DLS, Brookhaven, USA). Absorption spectra were recorded by UV-VIS-NIR spectro-photometer. The stability was evaluated by measuring the changed diameter in PBS buffer and serum at room temperature for up to 96 h. Long term stability was evaluated by measuring size of NPs before lyophilizing in 10 wt% sucrose solution and after resuspension. The amount of IR780 released from the IR780@RBC and IR780@rRBC in PBS buffer was studied using the dialysis method.

### Photothermal effect of IR780@rRBC

The obtained solutions of water, free IR780, IR780@RBC and IR780@rRBC were irradiated with NIR laser (808 nm, 1 W/cm^2^), and the temperature was recorded every 10 s for 180 s using both a temperature detector and an IR camera. The thermogenic capacity of above solutions was determined by repeated exposure to laser irradiation. Three cycles of irradiation were performed and the changed temperatures were recorded (808 nm, 1 W/cm^2^, 4 min of each time).

### Cytotoxicity

The cytotoxicity of IR780@rRBC NPs in CT-26 cells was measured according to a standard MTT assay at different incubation time. Briefly, the cells were seeded in 96-well plates (1 × 10^4^ cells per well) and cultured until reaching ∼80 % confluence. Then, the culture medium was replaced with a fresh medium containing different concentration of IR780, IR780@RBC or IR780@rRBC NPs, after incubation for 2 h, 8 or 24 h, the cell viability was measured by MTT assay at 490 nm. For photothermal toxicity, the laser irradiation (1 W/cm^2^, 5 min) was performed to each well (8 or 24 h incubation) at 808 nm for one time, and then the cell viability was measured by MTT assay at 490 nm.

### Macrophage cell uptake studies of IR780@rRBC NPs

To compare the stealth functionality of IR780, IR780@RBC and IR780@rRBC NPs, mouse macrophage cell line RAW264.7 was chosen for cell uptake studies. Simply, the cells were seeded into 6-well plates (2 × 10^5^ cells per well) in DMEM with 10 % FBS and 1 % penicillin and streptomycin. After cells were reaching ∼60 % confluence, they were washed by PBS and replaced with fresh complete cell culture medium containing IR780, IR780@RBC or IR780@rRBC NPs (1.56 µg/mL for IR780) for 8 h incubation. Then, cells were washed with PBS and fixed with 4 % paraformaldehyde for 30 min. After fixation, cells were washed with PBS and stained with DAPI for 10 min. Finally, cells were washed with PBS again and imaged by confocal microscopy.

### In vivo pharmacokinetics studies

Six-week-old female balb/c mice were used in compliance with protocol approved by the Institutional Animal Care and Use Committee of Nantong University. For pharmacokinetics studies, fifteen male mice were randomly divided into free IR780, IR780@RBC and IR780@rRBC NPs groups (n = 5). The above formulations were administered via intravenous injection with the IR780 dose of 1.4 mg/kg. At different time points (0, 15 min, and 1, 2, 4, 8, 12, 24, 48, 72, 96 h), blood samples were collected by retro-orbital bleeding. The content of IR780 in the serum samples was measured using a Varioskan Flash Spectral Scanning multimode plate reader (Thermo Fisher Scientific, Waltham, MA, USA).

### In vivo biodistribution and PTT therapeutic efficacy studies

For pharmacokinetics studies, 1 × 10^7^ CT-26 cells were subcutaneously injected into the right flank of each mouse. Two weeks later, the developed tumor was excised and cut into ∼1 mm^3^ pieces. The pieces of tumors were implanted into the right flank to establish subcutaneous tumors for the biodistribution and therapeutic studies.

Nine mice bearing xenograft tumor were used and divided into three groups (n = 3 per group) and were given a single dose of IR780@rRBC NPs (0.3 mg/kg IR780) by intravenous injection. Whole-body fluorescence imaging was performed using IVIS Lumina imaging system (Xenogen Co., USA) with excitation/emission set to 745/808 nm. Fluorescence images were acquired in anesthetized animals at different time points (12 h, 24 and 48 h). IVIS Living Imaging Software was used for the analysis of the amount of IR780 in tissues.

Therapeutic study started when the tumors reached ∼200 mm^3^.The mice were divided into groups (n = 6 per group) and treated with (i) PBS, (ii) IR780 + NIR laser, (iii) IR780@RBC + NIR laser, (iv) IR780@rRNC NPs, (v) IR780@rRNC NPs + NIR laser. All samples were administered via Intravenous injection (1.4 mg/kg IR780). The day of administration was designated as day 0. After 24 h, the tumors were exposed to the NIR laser irradiation (1 W/cm^2^) for 3 min [28]. The temperature change of the tumor was recorded and imaged using a visual IR camera (Fluke Corporation) at different time points for total 250 s. Treatment administrations and laser irradiation were repeated on days 2 and 3. Tumor sizes and mouse body weights were recorded every other day. Tumor volume (V) was calculated as V = d^2^ × D/2, where D and d are the longest and shortest diameter of the tumor, respectively.

### Statistical analysis

Statistical assessment was conducted by two-sided Student’s *t* test for two groups and one-way ANOVA analysis of variance for multiple groups (*P* < 0.05 was considered statistically significant). All analyses were performed with SPSS 19.0 for Windows.

## Results and discussion

### Preparation and characterization of IR780@RBC and IR780@rRBC

**Fig. 2:**
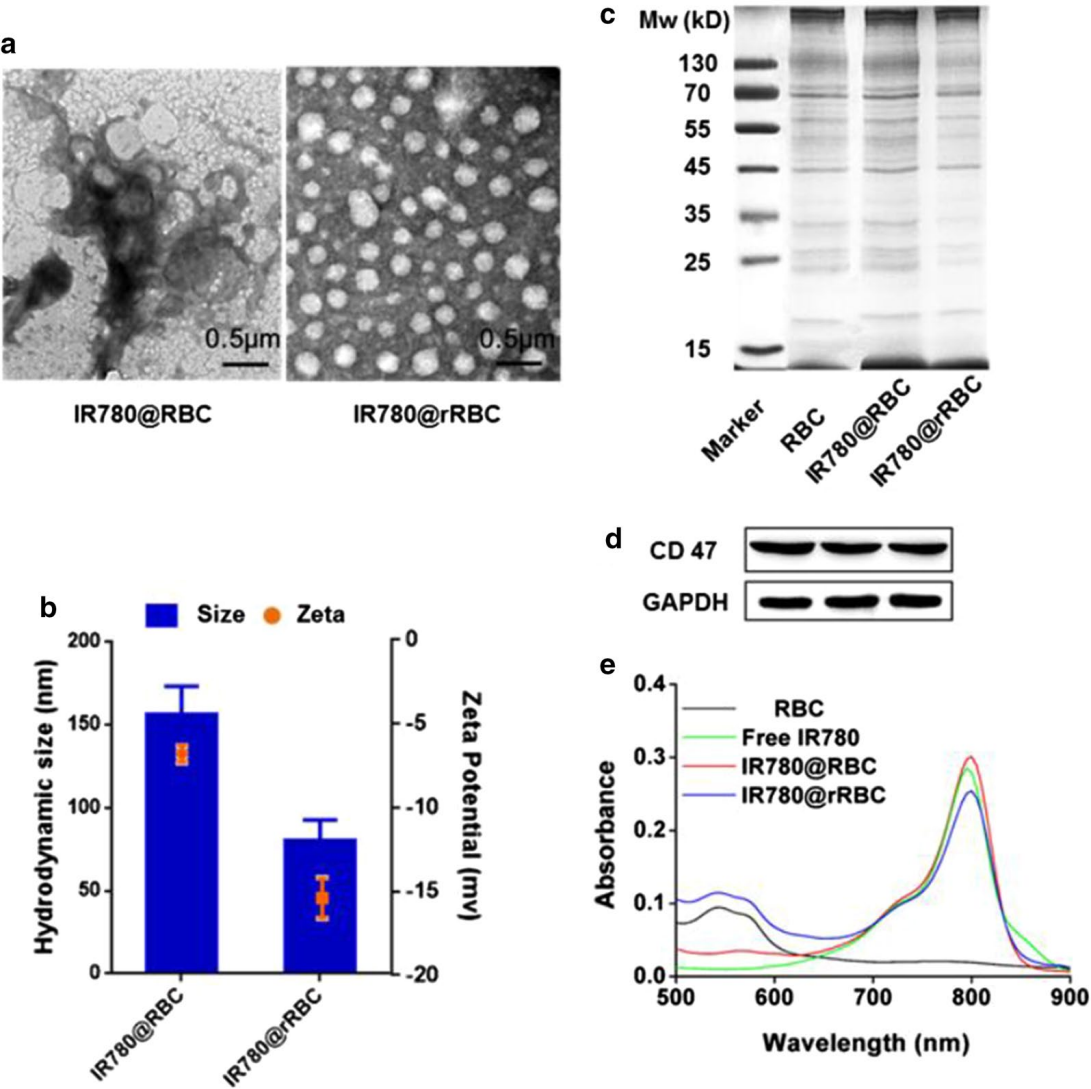
Characterization of IR780@RBC and IR780@rRBC NPs. **a** TEM images of IR780@RBC and IR780@rRBC NPs; **b** Size and zeta potential of IR780@RBC and IR780@rRBC NPs; **c** SDS-PAGE of natural RBC membrane, IR780@RBC and IR780@rRBC NPs; **d** CD 47 expression of natural RBC membrane, IR780@RBC and IR780@rRBC NPs by western blot; **e** UV-VIS-NIR absorption spectra of natural RBC membrane, free IR780, IR780@RBC and IR780@rRBC NPs

Several studies have reported that IR780 is tended to integrate with the hydrophobic parts of other components based on its strong hydrophobicity [23]. Hence, IR780 was easily enclosed in the hydrophobic area of RBC membranes to form IR780@RBC NPs by an ultrasonic method. For the preparation of IR780@rRBC NPs, a standard film dispersion method was used to encapsulate IR780 with the isolated and dehydrated lipids in ethanol, and then the PBS buffer containing endogenous proteins were adding to form NPs after forming the film. To characterize the reconstituted IR780@rRBC NPs, the morphology and particle size of the free IR780, IR780@RBC and IR780@rRBC were first measured by TEM and DLS. As shown in Fig. 2a, b, TEM images showed that IR780@rRBC NPs were more uniform and spherical compared with IR780@RBC NPs, and DLS results showed that the particle size of IR780@RBC and IR780@rRBC NPs was 156.4 ± 16.8 and 80.28 ± 12.4 nm, respectively. The inconsistent with the hydrodynamic diameter of IR780@RBC NPs obtained from TEM and DLS might be due to the increased IR780 concentration in the TEM dry process.

To analyze the overall protein content of the IR780@RBC and IR780@rRBC NPs, SDS-PAGE was used to run nature RBC membrane, IR780@RBC and IR780@rRBC nanoparticle formulations, followed by Coomassie staining for visualization. As shown in Fig. 2c, compared with RBC membrane, both nanoparticles had protein profiles that largely mirrored that of the corresponding membrane. Additionally, CD47, a specific immunomodulatory protein marker responsible for immune escape property in RBC membrane, was carried out by western blot and the results showed that a near equivalent degree could be maintained after the formation of IR780@RBC and IR780@rRBC NPs (Fig. 2d). These results here indicate that our biomimetic green strategy of “disassembly-reassembly” can bestow RBC surface proteins onto IR780@RBC and IR780@rRBC NPs for expected long-circulating delivery.

The absorption spectrum of IR780@RBC and IR780@rRBC NPs in Fig. 2e showed that IR780 was successfully incorporated into the RBC and rRBC membranes. The fluorescence spectra indicated that the distribution of IR780 was more uniformly via encapsulation into RBC membranes-based nanoparticles compared with free IR780 and IR780@RBC, because they had relatively little fluorescence quenching in PBS solution induced by unfavorable aggregation (Additional file 1: Figure S1). Encapsulation efficiency of IR780 in IR780@RBC and IR780@rRBC NPs was determined. The results showed that efficient loading with a high encapsulation efficiency of 75.2 ± 3.5 % and 91.4 ± 6.4 % was achieved, and the relative IR780 concentration in IR780@RBC and IR780@rRBC NPs was 750.2 and 910.4 µg/mL, respectively. Compared with the low solubility of free IR780 (0.4 µg/mL) in water [23, 29], the water solubility of IR780 was increased 1876-fold via encapsulation into RBC membranes-based nanoparticles, and its water solubility was further increased 2276-fold via encapsulation into rRBC membrane-based nanoparticles. Additionally, the digital pictures of the IR780@rRBC NPs in water, PBS and 10 wt% sucrose further confirmed their good solubility (Additional file 1: Figure S2). Taken together, these characterization data give strong physical evidence for successful loading IR780 into NPs wherein containing the properties of RBC membranes.

### In vitro stability and drug release of IR780@RBC and IR780@rRBC

**Fig. 3:**
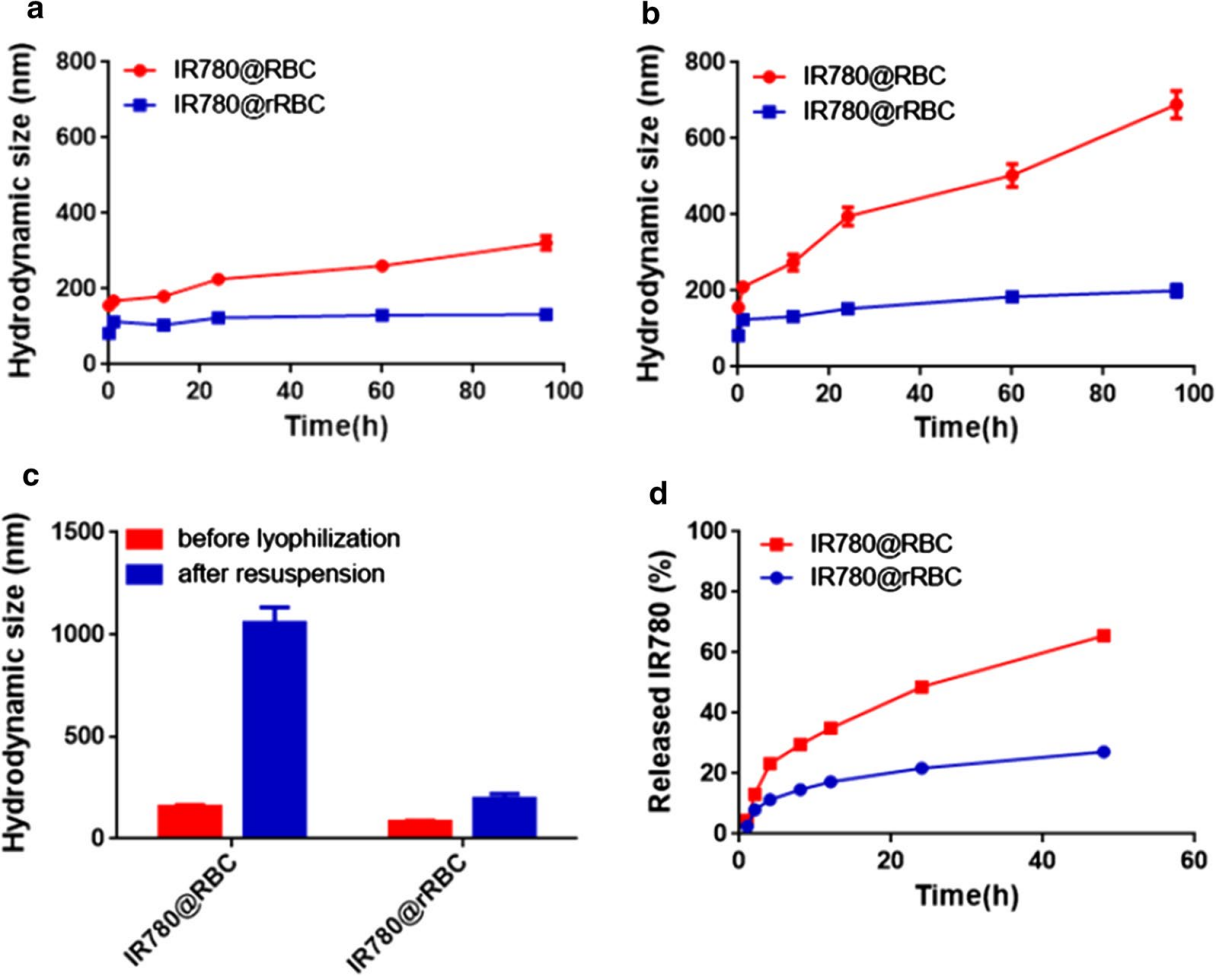
Nanoparticle stability. Z-average size of IR780@RBC and IR780@rRBC in PBS **a** and serum **b** over 96 h. **c** Z-average size of IR780@RBC and IR780@rRBC NPs before lyophilization in 10 wt% sucrose and after resuspension. **d** The release property of IR780@RBC and IR780@rRBC NPs in PBS buffer

Considering the different preparation strategies of these two nanoparticles, a set of assays were carried out to assess nanoparticle stability in various media. To determine stability of the IR780@RBC and IR780@rRBC NPs in solution over time, samples were stored in either phosphate buffered saline (PBS, pH 7.4) or 100 % serum, and their sizes were measured over time. The IR780@rRBC NPs exhibited stable size over a 96 h duration of the study in two media, while IR780@RBC NPs immediately aggregated in PBS to ~320 nm and in serum to ~700 nm (Fig. 3a, b). Regarding long term storage, the IR780@rRBC NPs formulation exhibited near identical size both before freeze-drying and after resuspension (Fig. 3c). In vitro release profiles of IR780 in PBS buffer were shown in Fig. 3d. The cumulative release of IR780 from IR780@rRBC NPs was less than 20 % during 48 h, showing that IR780 was stably entrapped in the IR780@rRBC NPs with low leakage in a simulated physiological environment. In contrast, the cumulative release of IR780 from IR780@RBC NPs could be reached 70 % during 48 h. Based on the improved stability and sustained release profile, we expect that IR780@rRBC NPs will decrease the toxicity and increase the tumor accumulation of IR780 in the following experiments.

### In vitro photothermal, cytotoxicity and cellular uptake of IR780@RBC and IR780@rRBC

**Fig. 4:**
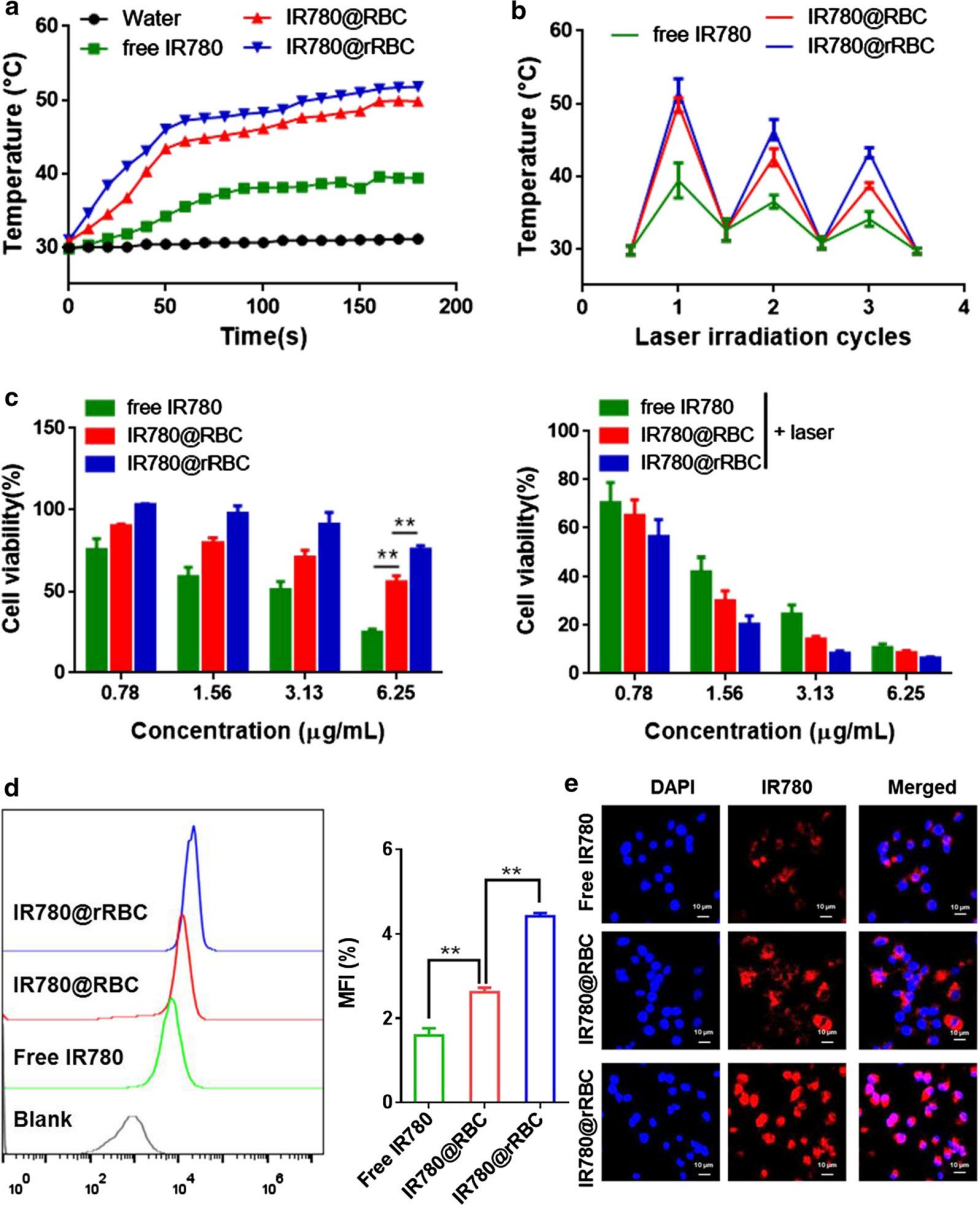
In vitro photothermal and cytotoxicity. **a** Solution temperatures of different formulations with the laser irradiation (n = 3). **b** Thermal curves of different formulations after repeated laser irradiation (n = 3). **c** Cell viability of different formulations on CT26 cells at 24 h incubation with or without laser. Cellular uptake of different formulations determined by flow cytometry d and confocal microscopy **e** on CT26 cells for 8 h incubation

To assess the photothermal effect of IR780@RBC and IR780@rRBC NPs, the temperature was recorded by incubating them with the laser irradiation (1 W/cm^2^) for 3 min and the repeated irradiation for three cycles (1 W/cm^2^). From Fig. 4a, b, the temperature of free IR780 increased from ~ 30 ℃ to ~ 40 ℃ and decreased to ~ 35 ℃ in the third cycle. In contrast, IR780@RBC and IR780@rRBC NPs exhibited a higher temperature increase form ~ 30 ℃ to ~ 50 ℃ and slowed temperature decrease form ~ 30 ℃ to ~ 40 ℃ in the third cycle. Thus, these results demonstrated that the formation of nanoparticles could improve the photothermal properties of IR780, which might be due to the decreased degradation rate of IR780 in NPs. In addition, the photothermal effect of IR780@rRBC was also confirmed using an IR camera (Additional file 1: Figure S3). These results are consistent with previous reports of encapsulation IR780 into nanostructured lipid carriers [22].

The cytotoxicity of IR780@RBC and IR780@rRBC NPs was then evaluated in CT-26 cells according to a standard MTT assays. As shown in Fig. 4c (Additional file 1: Figure S2), the order of toxicity without laser irradiation was as follows: free IR780 > IR780@RBC NPs > IR780@rRBC NPs, which reflected in the viability of cells treated with free IR780 at 6.25 µg/mL for 24 h declined to 25.07%, while the cell viability of IR780@RBC NPs and IR780@rRBC NPs could be maintained at 55.91 and 75.48 %, respectively. In contrast, the order of toxicity with laser irradiation was as follows: IR780@rRBC NPs > IR780@RBC NPs > free IR780, which reflected in the viability of cells treated with free IR780 at 3.13 µg/mL for 24 h decreased to 24.62 %, while the cell viability of IR780@RBC NPs and IR780@rRBC NPs could be achieved at 14.29 and 8.49 %, respectively. Furthermore, their cellular uptake was determined by flow cytometry and confocal microscopy, and the results indicated that the internalization of IR780@rRBC was higher than IR780@RBC and free IR780 after 8 h incubation (Fig. 4d, e). The above results demonstrated that IR780@rRBC and IR780@RBC NPs were low toxic compared with free IR780 before the laser irradiation due to their sustained release effect, although their cellular uptake were higher compared with free IR780. However, their photothermal toxicity was enhanced after the laser irradiation due to the burst release induced by membrane rupture when compared with free IR780.

### In vivo characterization of IR780@RBC and IR780@rRBC

**Fig. 5:**
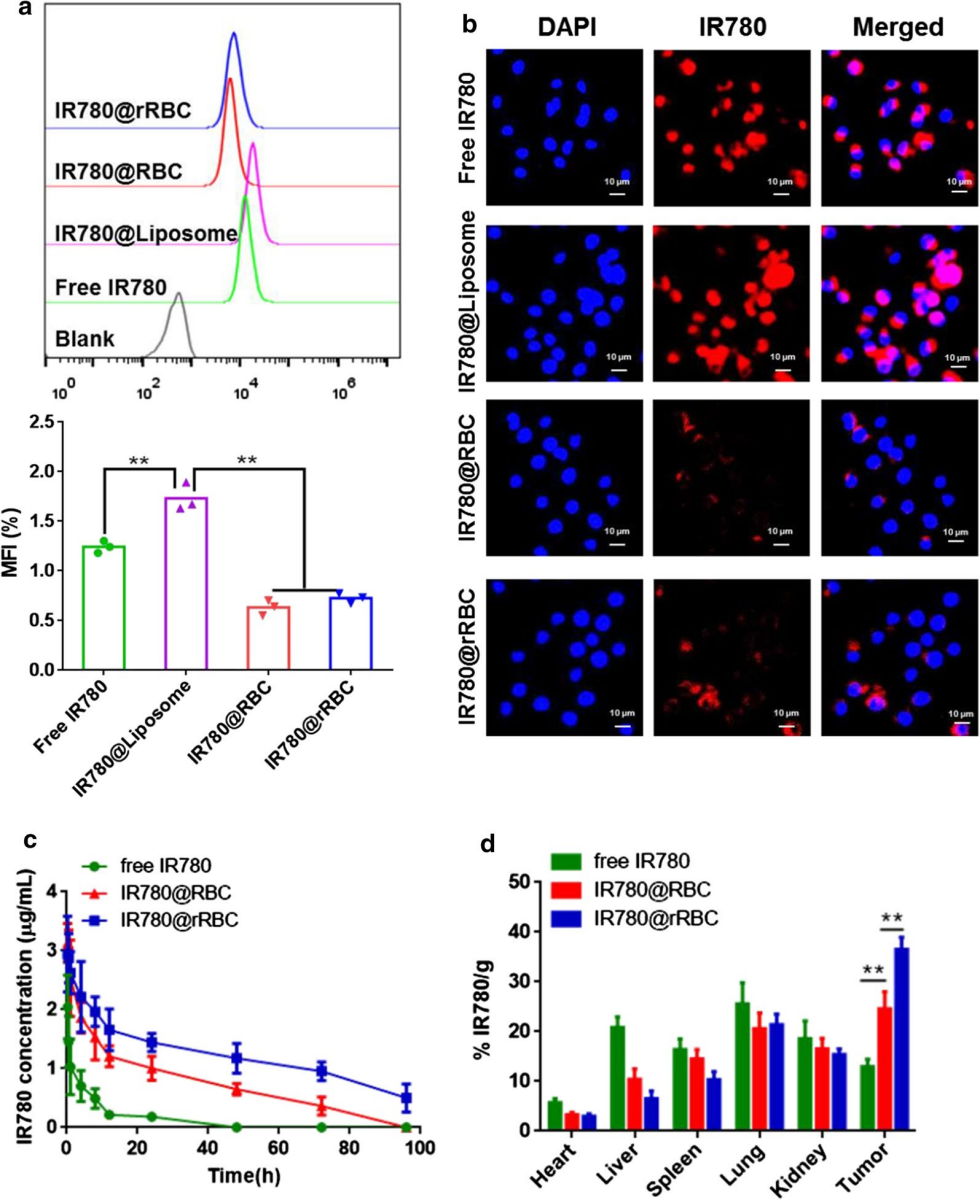
In vivo characterization. Cellular uptake of different formulations determined by flow cytometry **a** and confocal microscopy **b** on macrophage cells (Raw264.7) for 8 h incubation. **c** Blood circulation time of free IR780, IR780@RBC and IR780@rRBC NPs over a span of 96 h. **d** Biodistribution of free IR780, IR780@RBC and IR780@rRBC NPs 24 h after intravenous administration. All values are expressed as Means ± SD (n = 4)

After confirming the superior stability and photothermal effect of IR780@rRBC NPs compared with free IR780 and IR780@RBC NPs, we investigated whether IR780@rRBC NPs could inherit perfectly the stealth properties of RBC membrane, which reflected in effective escape in macrophage cells, prolonged circulation time and improved tumor accumulation. To assess these, the macrophage uptake study was first used to evaluate the ability of IR780@rRBC NPs to escape the capture of macrophage cells. Additionally, IR780@Liposome system was also prepared as a control group for comparing the macrophage escape ability. As shown in Fig. 5a, b, compared with free IR780, the IR780@RBC and IR780@rRBC NPs significantly reduced the macrophage uptake and exhibited the weaker NIR fluorescence in RAW264.7 cells. In contrast, the IR780@Liposome exhibited enhanced cellular uptake due to the affinity between lipids and cell membranes. These results indicated that the IR780@RBC and IR780@rRBC NPs inherited the immune system evade ability, and had the potential to escape the endothelial system clearance for further in vivo applications.

One of the outstanding advantages for erythrocytes as delivery system is to extend the circulation time based on a variety of immunomodulatory markers expressed on its cell membrane [30, 31]. Hence, IR780@RBC and IR780@rRBC NPs were expected to have longer circulation time compared with free IR780, which might resulted in the enhanced accumulation in tumor via EPR effects. To test this, in vivo pharmacokinetics studies were conducted initially by administrating intravenously of free IR780, IR780@RBC and IR780@rRBC NPs in mice. The concentration of IR780 in sampled blood was evaluated at indicated time points, and our results (Fig. 5c and Additional file 1: Table S1) showed that the peak concentration (C_max_) of IR780@rRBC, IR780@RBC and free IR780 was 2.94, 3.13 and 2.04 µg/mL respectively. The area under the plasma concentration−time curve (AUC) was 1.77 and 11.37 times higher in case of IR780@rRBC than IR780@RBC and free IR780 (118.82 vs. 67.14 vs. 10.45 µg/mL*h). In comparison to the free IR780 or IR780@RBC, IR780@rRBC also exhibited significantly prolonged half-life in the plasma (t_1/2_) from 49.74 to 34.74 h (IR780@RBC) or 7.98 h (free IR780). To analyze the bio-distribution, they were administered intravenously again, and the mice were sacrificed at 24 h after injection in order to collect the heart, liver, spleen, lung, kidneys and tumor for IR780 concentration analysis. As shown in Fig. 5d (Additional file 1: Figure S5), IR780@rRBC NPs exhibited dramatic enhancement of tumor accumulation. Additionally, IR780@rRBC NPs also demonstrated significantly decreased accumulations within certain major organs compared to IR780@RBC NPs. These data indicate that the IR780@rRBC NPs prepared by this controllable and green assembly strategy significantly improve the circulation time and tumor accumulation compared with free IR780 and IR780@RBC due to the nature properties of RBC membrane and the enhanced stability.

### In vivo photothermal therapy (PTT) of IR780@RBC and IR780@rRBC

**Fig. 6:**
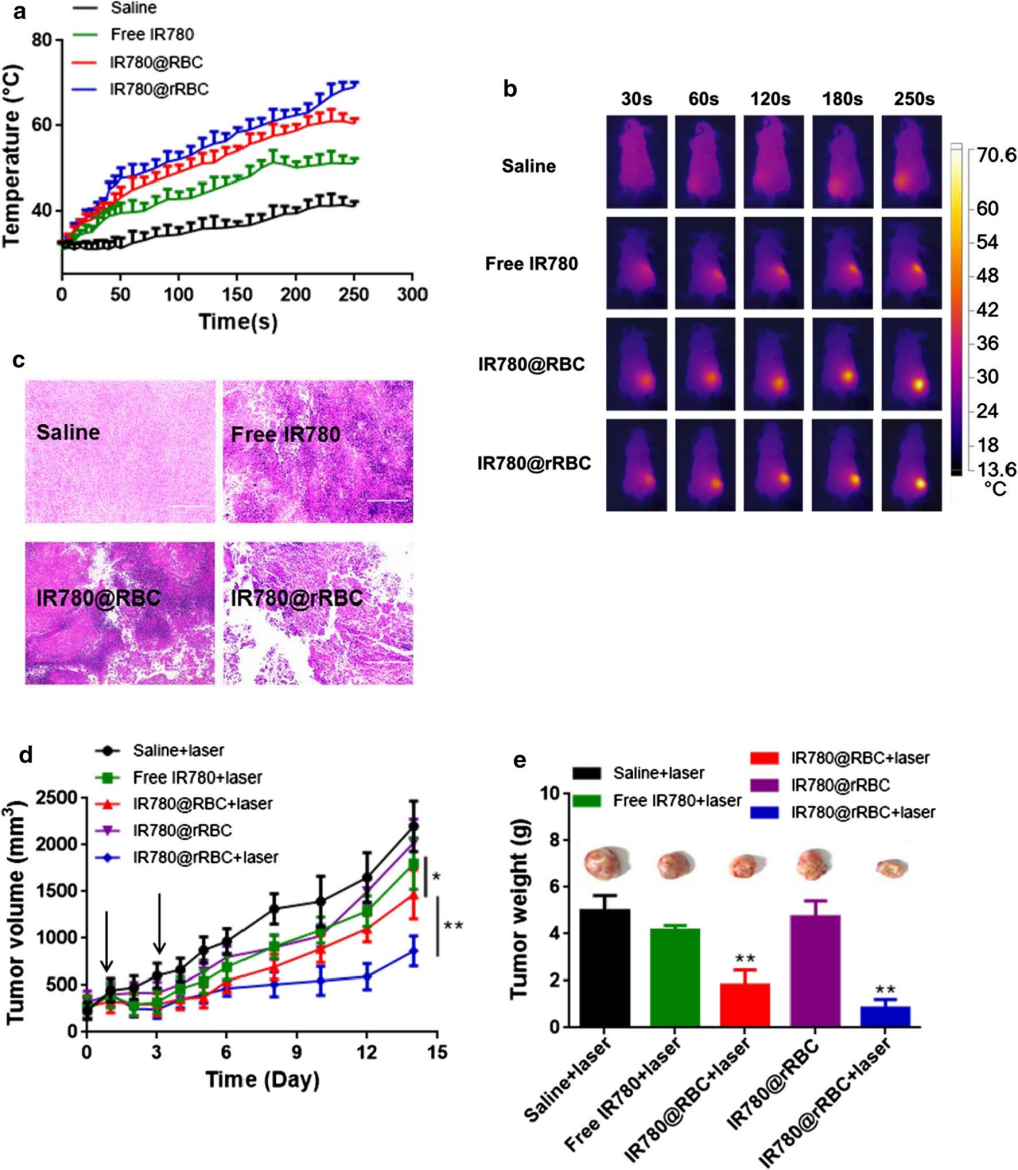
Antitumor PTT activity of IR780@RBC and IR780@rRBC following intravenous administration in CT-26 colon cancer model. **a** Tumor temperature changes after laser exposure. **b** The infrared thermal images of mice using IR camera. **c** H&E stained tumor sections after PTT. Scale bars, 200 μm. **d** Tumor volume after different formulations with or without laser. **e** Tumor weight and representative tumor photograph after different formulations with or without laser

Based on the improved circulation time and effective tumor accumulation of IR780@rRBC, we started to explore and compare the antitumor efficacy in tumor-bearing mice treated with saline, free IR780, IR780@rRBC and IR780@rRBC. First, we assumed that the accumulation of IR780@rRBC NPs would produce higher temperature in tumor site than IR780@rRBC and free IR780, and then the temperature curve was recorded in the tumor region 24 h after administration following the laser irradiation. As shown in Fig. 6a, b, the tumor temperature of IR780@RBC and IR780@rRBC NPs could reach ~ 60 ℃ and ~ 70 ℃ respectively, while the free IR 780 and saline groups showed slightly temperature increase to ~ 50 ℃ and ~ 40 ℃ respectively under the same laser conditions. Such a high temperature increase treated by IR780@RBC and IR780@rRBC NPs in the local tumor was expected to be sufficient to damage the tumor cells. H&E results exhibited significant necrosis of the tumors in the IR780@RBC and IR780@rRBC groups but not in any of the saline group (Fig. 6c). Antitumor efficacy of the photothermal therapy with IR780@rRBC was then studied and the mice were illuminated with laser irradiation at 24 and 72 h post-injection. As shown in Fig. 6d, e, combination of laser treatment with IR780@RBC and IR780@rRBC significantly suppressed the tumor volume compared with saline and free IR780 groups, and the antitumor efficacy of IR780@rRBC was better than that of IR780@RBC treatment. Statistical results of tumor weight further confirming the superiority of IR780@rRBC NPs prepared by our reported novel assembly biomimetic technology.

Finally, we evaluated whether treatment with the IR780@rRBC NPs possessed any potential toxic side effects (Additional file 1: Figure S6a–c). Body weight change was used to detect the overall treatment-induced toxicity and no obvious weight changes were observed in any of the groups. ALT (alanine aminotransferase), AST (aspartate aminotransferase) for hepatic function, and UREA (urea nitrogen) and CREA (creatinine) for renal function, were evaluated in blood samples and no visible differences of the above enzymes could be find in saline and IR780@rRBC treated group. H&E staining of the main organs (heart, liver, spleen, lung, and kidney) further confirmed safety of the treatment with IR780@rRBC NPs, as there were no apparent pathological changes compared with saline group. Overall, these findings suggest that IR780@rRBC NPs is a safe therapeutic formulation in photothermal anticancer therapies.

## Conclusions

In the present work, we reported a green “disassembly-reassembly” strategy to construct biomimetic reconstituted RBCs membrane (rRBCs) whose composition comes from the endogenous proteins and lipids from nature RBC membrane. IR780, a NIR dye, was used as pattern drug to be loaded in rRBCs to form IR780@rRBC NPs for demonstrating the benefits and feasibility of rRBCs with IR780@RBC NPs and free IR780 as controls. The properties of IR780@rRBC NPs, including decreased toxicity and increased stability in vitro, prolonged circulation capacity and enhanced PTT therapeutic efficiency in vivo, are improved, which may be due to the uniform distribution of IR780 in rRBC NPs through “disassembly-reassembly” strategy. These results indicate that the reconstituted rRBCs have great potential to replace nature RBCs for developing RBC membranes derived nanoparticles. Additionally, the endogenous lipids can also be replaced or supplied by other functional lipids. Notably, when the endogenous proteins are reconstituted into the rRBC in the reassembly process, some proteins may be encapsulated into the aqueous core of rRBC. Therefore, the main functional proteins in reconstituted nanoparticles need to be determined for ensuring the endowed abilities, such as CD47 for RBCs. Overall, rRBCs biomimetic represents a controllable and versatile RBC-inspired delivery platform compared with chemical synthesis and physical coating technology.

## Supplementary Information

**Additional file 1. **Additional figures and table. **Figure S1.**The fluorescence spectra of free IR780, IR780@RBC and IR780@rRBC in PBS and ethanol solutions. **Figure S2.**The digital pictures of the IR780@rRBC NPs in water, PBS and 10wt% sucrose. **Figure S3.**IR images of water, free IR780, IR780@RBC and IR780@rRBC after 808-nm laser irradiation for 180s. **Figure S4.**Cell viability of different formulations on CT26 cells at different incubation time with or without laser (2h, 8h). **Figure S5.**The representative organ images of free IR780, IR780@RBC and IR780@rRBC NPs in biodistribution study. **Figure S6.**In vivo toxicity. a) Body weight changes over the treatment period. b) Table of serum biochemical indicators acquired from mice on day 14. c) H&E staining of the heart, liver, spleen, lung, and kidney from mice on day 14.**Table S1.**Pharmacokinetic parameters for i.v. injection in mice.

## Data Availability

The datasets used and analyzed during the current study are available from the corresponding author on reasonable request.

## Abbreviations

RBC: Red blood cell
NPs: Nanoparticles
rRBCs: Reconstituted Red blood cell membranes
RLNs: Reconstituted lipoprotein nanoparticles
ALT: Alanine aminotransferase
AST: Aspartate aminotransferase
UREA: Urea nitrogen
CREA: Creatinine
