# Supplementary material for: Biomimetic recombinant of red blood cell membranes for improved photothermal therapy
Source: J Nanobiotechnology. 2021 Jul 18;19:213. doi: 10.1186/s12951-021-00949-7 (PMC8286575; doi:10.1186/s12951-021-00949-7)
Supplement: Supplementary file 1 — Additional file 1. Additional figures and table. Figure S1.The fluorescence spectra of free IR780, IR780@RBC and IR780@rRBC in PBS and ethanol solutions. Figure S2.The digital pictures of the IR780@rRBC NPs in water, PBS and 10wt% sucrose. Figure S3.IR images of water, free IR780, IR780@RBC and IR780@rRBC after 808-nm laser irradiation for 180s. Figure S4.Cell viability of different formulations on CT26 cells at different incubation time with or without laser (2h, 8h). Figure S5.The representative organ images of free IR780, IR780@RBC and IR780@rRBC NPs in biodistribution study. Figure S6.In vivo toxicity. a) Body weight changes over the treatment period. b) Table of serum biochemical indicators acquired from mice on day 14. c) H&E staining of the heart, liver, spleen, lung, and kidney from mice on day 14.Table S1.Pharmacokinetic parameters for i.v. injection in mice. [file 12951_2021_949_MOESM1_ESM.docx]

**Biomimetic Recombinant of Red Blood Cell Membranes for Improved Photothermal Therapy**

Pengkai Wu^1,2,#^, Xing Jiang^3,#^, Shuai Yin^1^, Ying Yang^1^, Tianqing Liu^4^, Kaikai Wang^1,5,*^

^1^School of Pharmacy, Nantong University, Nantong 226001, China

^2^Department of Hepatobiliary Surgery, the Affiliated Drum Tower Hospital of Nanjing University Medical School, Nanjing 210093, China

^3^College of Nursing, Nanjing University of Chinese Medicine, Nanjing 210029, China

^4^NICM Health Research Institute, Western Sydney University, Westmead, 2145, Australia

^5^Nantong Municipal Hospital of Traditional Chinese Medicine, Nantong 226001, China

^#^ These authors contribute equally

^*^ Corresponding author: kirk2008@126.com

**
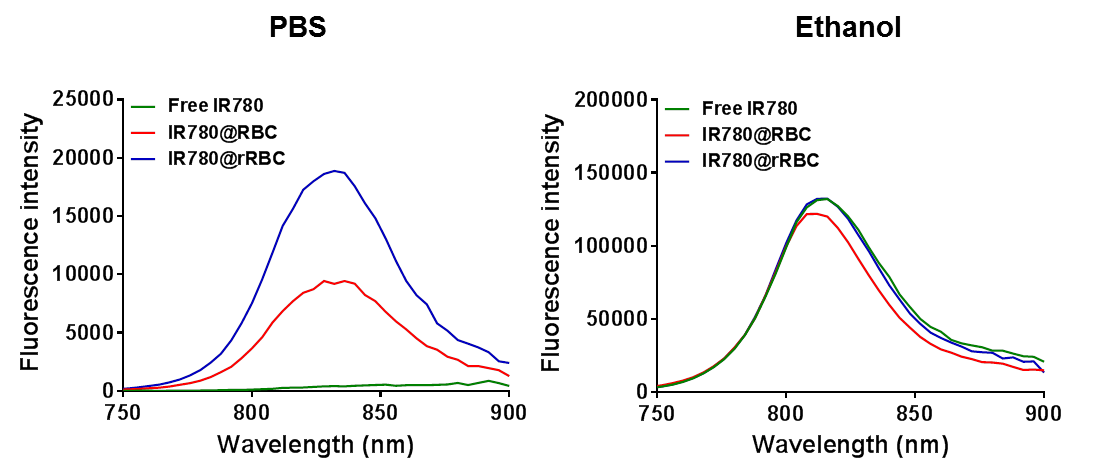
**

**Figure S1.** The fluorescence spectra of free IR780, IR780@RBC and IR780@rRBC in PBS and ethanol solutions.


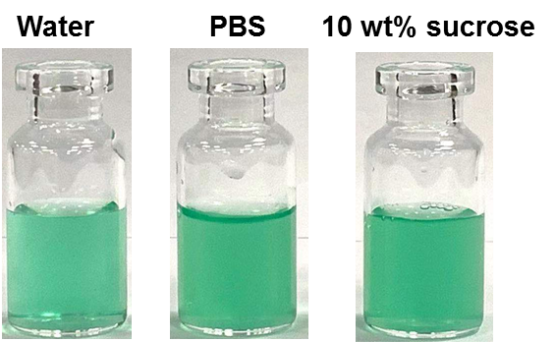


**Figure S2.** The digital pictures of the IR780@rRBC NPs in water, PBS and 10 wt% sucrose.


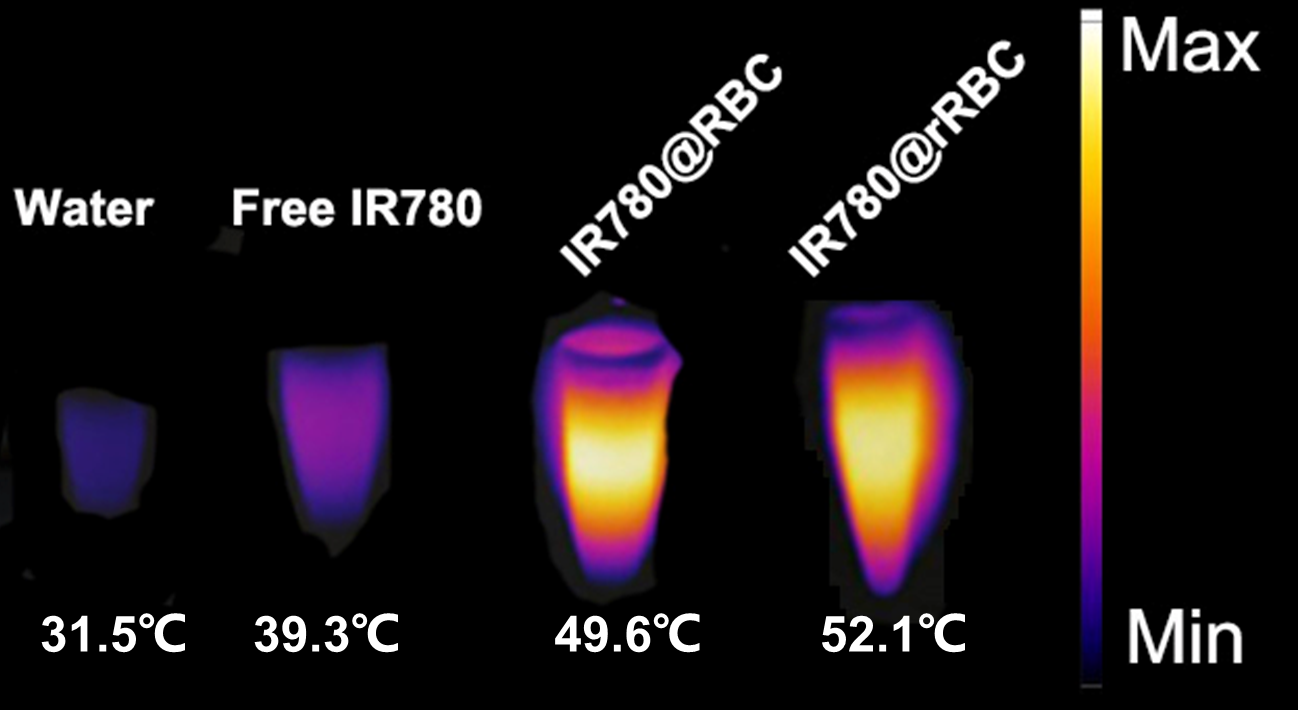


**Figure S3.** IR images of water, free IR780, IR780@RBC and IR780@rRBC after 808-nm laser irradiation for 180 s.


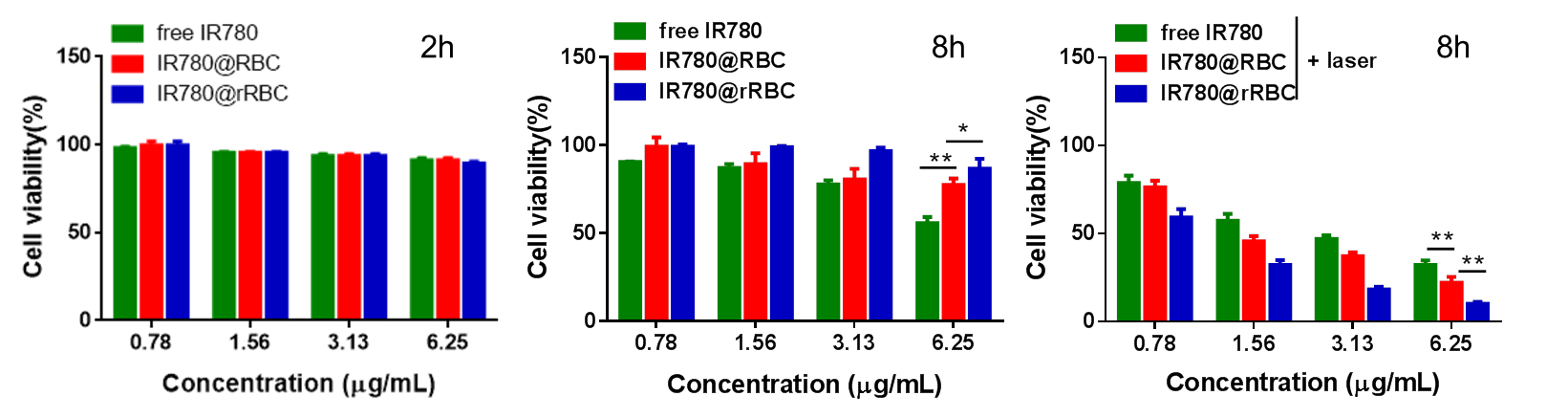


**Figure S4.** Cell viability of different formulations on CT26 cells at different incubation time with or without laser (2h, 8h).


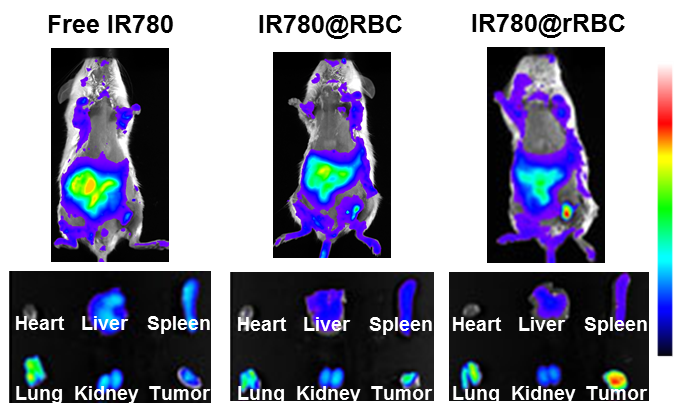


**Figure S5.** The representative organ images of free IR780, IR780@RBC and IR780@rRBC NPs in biodistribution study.


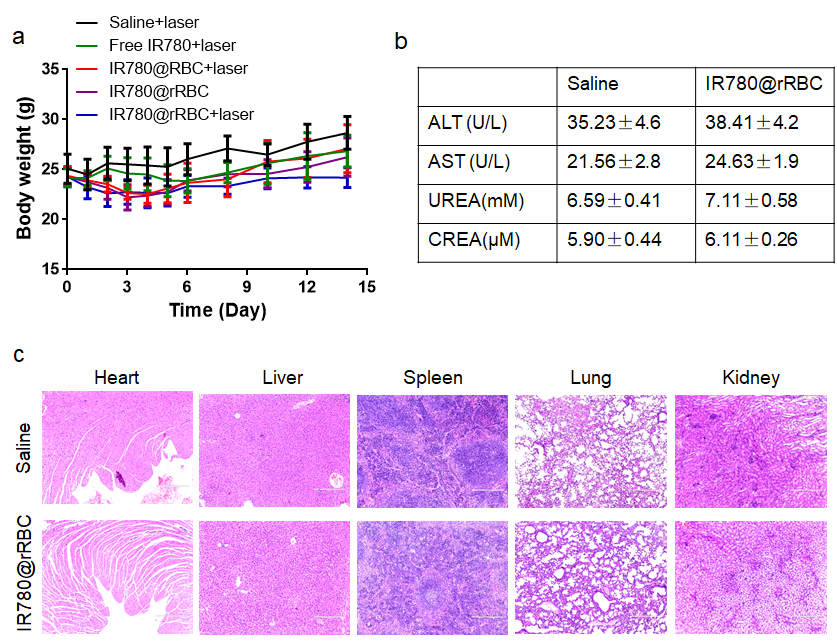


Figure S6. *In vivo* toxicity. a) Body weight changes over the treatment period. b) Table of serum biochemical indicators acquired from mice on day 14. c) H&E staining of the heart, liver, spleen, lung, and kidney from mice on day 14.

Table S1. Pharmacokinetic parameters for i.v. injection in mice. Cmax: Maximum concentration; AUC: Area under the curve; Tmax: Peak time; t_1/2_: Half-life time; MRT: Mean residence time. *P<0.05; **P<0.01, versus free IR780.

| Pharmacokinetic  Parameters | Free IR780 | IR780@RBC | IR780@rRBC |
| --- | --- | --- | --- |
| Cmax(µg/mL) | 2.04 | 3.13 | 2.94 |
| AUC (µg/mL*h) | 10.45 | 67.14 | 118.82 |
| Tmax (h) | 0.25 | 0.25 | 0.25 |
| t_1/2_ (h) | 7.98 | 34.74 | 49.74 |
| MRT (h) | 11.85 | 45.96 | 67.81 |
